# Biphasic remodeling of arcuate nucleus perineuronal nets in diet-induced obese mice

**DOI:** 10.1016/j.molmet.2026.102460

**Published:** 2026-10-15

**Authors:** Alexander Carbonell, Jarrad M. Scarlett, Ellie I. James, Kendra L. Francis, Kathryn Tyler, Tamim J. Chiba, Xavier Xong, Michael A. Haidar, Spoorthy Siddam, Aarun S. Hendrickson, Noah Marzouk, Cristiana J. Meuret, Caeley L. Bryan, Nina Brandt, Lauren E. Gilman, Jeremy M. Frey, Vladislav Baglaev, Mauricio D. Dorfman, David A. Tovar, Gregory J. Morton, Joshua P. Thaler, Michael W. Schwartz, Kimberly M. Alonge

**Affiliations:** 1 Department of Neuroscience, University of Florida, Gainesville, FL, USA; 2 McKnight Brain Institute, University of Florida, Gainesville, FL, USA; 3 University of Washington Medicine Diabetes Institute, University of Washington, Seattle, WA, USA; 4 Department of Pediatric Gastroenterology and Hepatology, Seattle Children's Hospital, Seattle, WA, USA; 5 Department of Medicinal Chemistry, University of Washington, Seattle, WA, USA; 6 Data Science Institute, Vanderbilt University, Nashville, TN, USA; 7 Department of Psychology, Vanderbilt University, Nashville, TN, USA

**Keywords:** Hypothalamus, Obesity, Diabetes, Perineuronal nets, Chondroitin sulfates, Extracellular matrices

## Abstract

The hypothalamic arcuate nucleus (ARC) contains neuronal populations crucial to the regulation of energy and glucose homeostasis. Many of these neurons are enmeshed by specialized extracellular matrix structures known as perineuronal nets (PNNs) that are comprised of chondroitin sulfate proteoglycans, including aggrecan (ACAN), and attached chondroitin sulfate-glycosaminoglycans. In mice, the effect of high-fat diet (HFD) to increase the abundance of ARC PNNs has been implicated in obesity- and diabetes-associated metabolic impairments. However, neither the biochemical nature nor the time course of these HFD-induced PNN changes is known. In this current work, we report that the response of ARC PNNs to HFD feeding is biphasic, characterized by a steady increase in abundance during the first ∼3 months, followed by a gradual decrease at later time points, as diet-induced obesity (DIO) becomes more fully established. Our findings suggest that this novel, biphasic patten of PNN remodeling is *1)* driven by weight gain rather than by HFD composition *per se*, *2)* specific to the hypothalamic ARC region, and *3)* parallels obesity-associated changes in the circulating glucose level. Furthermore, histochemical analysis reveals that the early increase of ARC PNNs is due primarily to changes in CS glycan attachments rather than the underlying proteoglycan core protein, while long-term HFD associates with reductions of both CS glycans and ACAN. This biphasic remodeling process becomes detectable with the onset of excess weight gain and is paralleled by obesity-associated changes in glycemia. Based on these findings, we conclude that in response to HFD feeding, ARC PNNs exhibit a degree of plasticity not seen in other brain areas and that dynamic PNN remodeling associates with changes in peripheral glycemia.

## Highlights

• Perineuronal nets in the arcuate nucleus (ARC) undergo biphasic remodeling in response to HFD feeding.
• ARC PNN abundance initially increases with HFD feeding, followed by a gradual decline as obesity becomes established.
• ARC PNN remodeling mirrors phasic responses in the circulating glucose level.

## Graphical abstract

**Figure:**
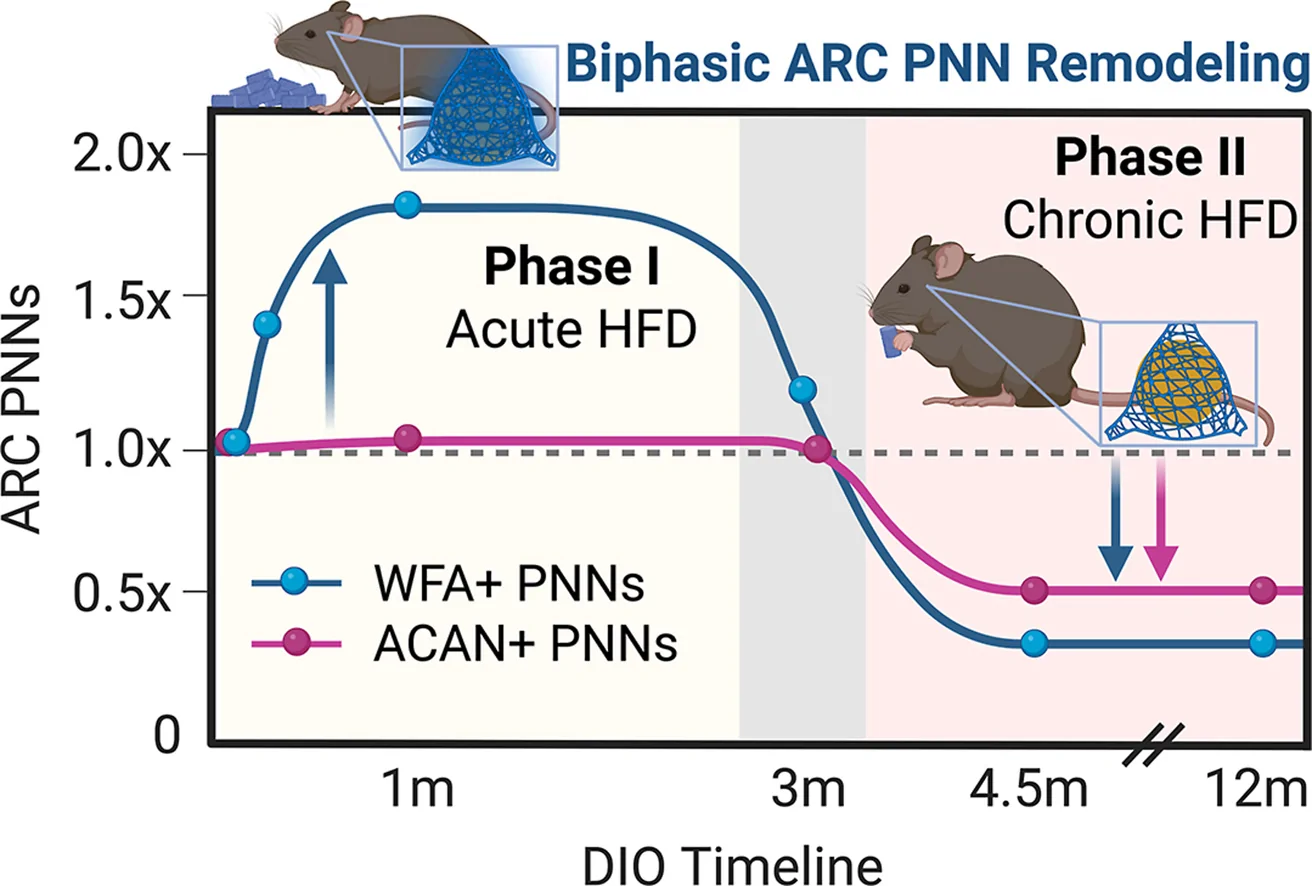

## 1 Introduction

Neurons located within the arcuate nucleus (ARC) of the mediobasal hypothalamus (MBH) transduce hormonal and nutritional cues into adaptive changes of energy balance and glucose metabolism [1]. Among these populations are GABAergic agouti-related peptide (AgRP) neurons that are enmeshed by unique extracellular matrix (ECM) structures termed perineuronal nets (PNNs) [2], [3], [4]. ARC PNN matrices are composed of chondroitin sulfate-glycosaminoglycans (CS-GAGs) attached to a heavily glycosylated aggrecan (ACAN) proteoglycan core protein [5], [6], [7], [8]. Although CS-GAGs can be detected using the lectin *Wisteria floribunda* agglutinin (WFA), antibody labeling of the ACAN protein backbone can be hindered by steric epitope masking and antibody charge repulsion resulting from the attached CS-GAGs. Functionally, PNNs contribute to neural-circuit organization and network connectivity by supporting elevated activity of the neurons that they enmesh [2], [9].

We recently demonstrated that ARC AgRP neurons are uniquely responsive to changes in peripheral glucose levels [10], and that in the *ob/ob* mouse model of obesity and type 2 diabetes (T2D), diabetic hyperglycemia can be normalized by silencing these neurons [11]. We have also shown that in the Zucker diabetic fatty (ZDF) rat model of type 2 diabetes (T2D), reduced PNN enmeshment of AgRP and other ARC neurons is implicated in the pathogenesis of hyperglycemia [8]. Conversely, increased ARC PNN abundance has been implicated in metabolic impairments associated with consuming a high-fat high-sucrose (HFHS) diet [6]. While these findings establish ARC PNNs as plastic and change in response to metabolic perturbations, the pathophysiological significance and directionality of these remodeling events remain a matter of some controversy. Specifically, whereas mice with diet-induced obesity (DIO) show increases in ARC PNN abundance [6], this effect has not been consistently observed across experimental models and protocols [4], [12]. To bring clarity to these discrepant findings, we sought in the current work to map the dynamics of ARC PNN remodeling induced by DIO.

To this end, we characterized temporal changes in ARC PNN abundance and composition in mice exposed to two different obesogenic diets, over time intervals ranging from 3 days to 12 months. Our findings reveal a biphasic pattern of ARC PNN remodeling: initially, the abundance of WFA+ PNNs enmeshing ARC neurons increases, reaching a plateau phase before gradually decreasing below its baseline value. This biphasic pattern appears to be driven more by initial weight gain than diet composition, and it tracks with long-term, obesity-associated changes in glycemia, consistent with a functional relationship between ARC PNN abundance and glycemic control. Biochemical analysis further shows the first phase of this ARC PNN remodeling process to be driven by changes in the CS glycans rather than the core protein, whereas the subsequent second phase loss of PNNs in late-stage DIO involves reductions of both core protein and CS-GAG attachments. Collectively, these findings establish ARC PNNs as metabolically responsive ECM structures that undergo distinct molecular and temporal phases of remodeling during DIO.

## 2 Materials and methods

### 2.1 Animals

For characterization of WFA+ and ACAN+ ARC PNNs, control mice (Jackson C57 B l/6 J strain #000664) were housed under specific pathogen-free conditions in a temperature-controlled room under 14 h:10 h light–dark cycle and had *ad-lib* access to water and PicoLab® Rodent Diet 20 chow (#5053) that contains calories provided by protein (24.5%), fat (13.1%), and carbohydrates (62.4%). In this study, two separate obesogenic dietary interventions were used, each of which was compared to chow controls: *1)* HFD (Research Diets, #D12492) that contains calories by protein (20.0%), fat (60.0%), and carbohydrates (20.0%), and *2)* HFHS (BioServ, #F4997) that contains calories by protein (14.9%), fat (58.8%), carbohydrates (26.3%), and has added cholesterol (0.15%).

#### 2.1.1 3 d, 7 d, 3 m, 4.5 m, and 12 m DIO wild type cohorts

For acute (3 d and 7 d) HFD feeding studies, single-housed, male C57 B l/6 J mice (9wk of age) were placed on either chow or HFD for either 3 or 7 days (3 d, n = 7 chow and n = 8 HFD mice; 7 d, n = 8 mice/group). For chronic (3 m–12 m) HFD feeding studies, the 3 m HFD feeding study utilized single-housed male mice that were placed on either chow or HFD (n = 4 chow mice, n = 4 HFD mice) starting at 11wk of age and sacrificed 12wk later. The 4.5 m DIO study utilized group-housed, male mice that were placed on either chow, HFD, or HFHS (n = 10 mice/group) starting at 9wk of age and sacrificed 18wk later. The 12 m DIO study utilized group-housed male mice that were placed on chow or HFD (n = 9 chow mice, n = 9 HFD mice) starting at 9wk of age and sacrificed 48wk later. Body composition analysis of body fat mass and lean mass for the 4.5 m DIO cohort was determined using quantitative magnetic resonance spectroscopy (EchoMRI 3-in-1; Echo MRI, Tx) in live conscious mice with support from the NIDDK-funded Nutrition Obesity Research Center (NORC) Energy Balance Core at the University of Washington (*data not shown*) [11].

#### 2.1.2 28 d wild type and *db/db* cohorts

The 28 d DIO studies included both wild-type and *db/db* mice (Jackson B6·BSK(D)-*Lepr*^*db*^*/*J strain #000664). Four separate cohorts of group-housed male mice were studied, two of which remained on chow while the other two were placed on the HFD (n = 7 chow wild type mice, n = 8 HFD wild type mice, n = 8 chow *db/db* mice, n = 7 HFD *db/db* mice) starting at 11wk of age and sacrificed 28 d later.

### 2.2 Fluorescent labeling of PNNs

#### 2.2.1 Tissue processing

For immunofluorescence studies, mice were anesthetized with ketamine and xylazine overdose and cardiac perfused with 0.1 M phosphate buttered saline (PBS) followed by 4% paraformaldehyde (PFA) in 0.1 M PBS. The brains were then extracted, post-fixed in 4% PFA for an additional 48 h at 4 °C, cryopreserved in 30% sucrose in 0.1 M PBS, and frozen in optimal cutting temperature (OCT) compound on dry ice. Long-term storage occurred at −80 °C, brains were acclimated at −20 °C for 2 h prior to cryosectioning, the hypothalamus was exhaustively sectioned with a Leica CM1950 cryostat at 30−μm thick coronal sections, and sections were stored as free-floating in 12-well dishes containing 0.1 M PBS +0.02% sodium azide at 4 °C until processed for immunofluorescence.

#### 2.2.2 ChABC pre-digestion time course

Because attached CS glycans can obscure immunofluorescence detection of the underlying proteoglycan core protein, including aggrecan, we sought to remove the glycans by enzyme digestion prior to antibody labeling. To this end, we optimized the duration of incubation with Chondroitinase ABC (ChABC) to remove glycans from brain tissues prior to immunofluorescence labeling. To account for differences in ARC PNN changes in response to chow *vs.* HFD, we serially sectioned n = 1 male mouse fed chow (in-house C57/B6 wild type, 1 year of age) and n = 1 male mouse fed HFD (C57/B6 wild type, 20wk of HFD, sacrificed at 6 m of age). Coronal slices selected for the study were evenly distributed between Bregma position −1.6 mm and-1.9 mm as technical replicates, containing 3–4 sections for each incubation time point from each mouse.

We first established a time course for PNN digestion using tissues from chow-fed mice. Tissue sections were initially washed 3x in sterile 50 mM ammonium bicarbonate (pH 7.6) followed by a 0–24 h incubation at 37 °C in 0.5U/mL of ChABC (C3667, Millipore Sigma) in 50 mM ammonium bicarbonate. Histological results for each mouse were normalized to the 0 h (non-digested) section from each mouse individually as technical replicates. Optimal ChABC pre-digestion showed that while WFA+ PNNs were no longer detectable, the underlying ACAN labeling is sufficiently enhanced to enable quantitative assessment (Supplemental Fig. 2). After establishing the digestion time course in the chow mouse, we repeated the digestion procedure in tissues from DIO mice for comparison (Main Figure 3).

#### 2.2.3 Immunofluorescence and fluorescence analyses

Immunofluorescence (IF) was performed according to our published methods [3], [4], [8], [13], [14]. Briefly, free-floating sections underwent antigen retrieval in 10 mM trisodium citrate (pH 8) and were heated at 95 °C for 20 m. After cooling to room temperature (RT), tissue sections were permeabilized for 25 m at RT in 0.1 M PBS + 0.2% Triton X-100 and then blocked in 10% normal donkey serum (NDS, 017-000-121, Jackson ImmunoResearch) for 2 h at 37 °C in 0.1 M PBS + 0.05% Triton X-100 (PBS-T). Sections were transferred to primary aggrecan (ACAN) antibody (AB1031, Millipore, 1:250 dilution) or fluorescently labeled using a biotin-conjugated *Wisteria Floribunda* agglutinin lectin (L1516; Sigma–Aldrich, 1:1,000 dilution). After an overnight incubation, sections were washed with PBS-T, incubated for 2 h at RT in 1:1,000 secondary antibodies in PBS-T + 1% NDS, and then counterstained for DAPI. Tissues were then mounted, dried, and cover slipped using Floromount-G (4958–02, ThermoFisher).

#### 2.2.4 Imaging and quantitation

To quantify both WFA+ and ACAN+ PNN abundance, both the percent area (%A) and mean fluorescence intensity (MFI) were measured using our previously established method for the assessment of hypothalamic PNNs [8]. First, images were captured on either a Keyence BZ-X800 fluorescence microscope (10X) (Itasca, Illinois) or a Nikon A1R HD confocal (10X and 3X zoom) (Melville, New York) using the same exposure settings across groups within the same study. ARC regions were defined as either −1.7, −1.8, or −1.9 mm from Bregma. In Fiji open-source imaging software [15], a constant background was first subtracted before the MFI (per mm2) and %A coverage (using a set threshold for each study and target individually) were determined. The averaged intensity for ARC PNNs across 2–3 ARC regions was computed and then normalized to the mean of the chow control values. Investigators remained blinded to group assignment during analysis.

### 2.3 Statistical analyses

For each DIO study, groups were matched for sex, age, body weight and blood glucose levels prior to the start of dietary intervention. Error bars represent the mean ± standard error of the mean (SEM). GraphPad Prism® 10 (GraphPad Software, Inc., La Jolla, CA) was used for statistical analyses. Data with two groups were first analyzed for normality using Shapiro–Wilk test and normally (Gaussian) distributed data were sequentially analyzed using parametric t-test (two-tailed); data that did not pass normality were instead assessed using non-parametric Mann Whitney test (two-tailed). All p-values, t-values (t), and degrees of freedom (df) are provided in Supplementary Tables 1–9. Studies comparing more than two groups used a one-way ANOVA with multiple comparisons and Tukey's post-test, or a 2-way ANOVA/mixed effects with Sidak's post-test; all p-values, degrees of freedom, and F-values are provided in the supplementary tables. All samples excluded due to histological processing errors were reported in the Supplementary Tables. Statistical analyses for Main Figure 1 and Supplementary Fig. 1 are shown in Supplementary Tables 1–4, Main Figure 2 are shown in Supplementary Tables 5–6, Main Figure 3 and Supplementary Fig. 2 are shown in Supplementary Table 7, and Main Figure 4 statistical analyses are shown in Supplementary Tables 8–9.

## 3 Results

### 3.1 HFD feeding rapidly increases ARC WFA+ PNN abundance

The abundance of ARC WFA+ PNNs was recently reported to increase after 28 d of HFHS (43% fat, 20% carbohydrate) treatment, whereas no changes were detected at shorter (*i.e.,* 3 d and 7 d) time points [6]. To better understand the time course of ARC PNN changes in response to another DIO model, we quantified changes in the abundance of ARC WFA+ PNNs following 3 d, 7 d, or 28 d on a standard HFD (60% fat, 20% carbohydrate). 3 d after switching to the HFD, neither body weight (g) (Figure 1A) nor blood glucose (Figure 1B) differed from chow-fed controls, as expected. By Day 7, however, body weight had begun to increase (Figure 1D), elevating further by Day 28 (Figure 1G) of HFD feeding. Daily blood glucose measurements revealed that glycemia increased in parallel with the progressive increase in body weight (Figure 1E,H). This time course was also parallelled by changes in ARC WFA+ PNNs (quantified by both mean fluorescence intensity (MFI) and percent area (%A)). Thus, while no significant changes in PNN abundance were observed on Day 3 (Figure 1C), PNN abundance increased significantly by Day 7 (Figure 1F) and values were further elevated by Day 28 (Figure 1I) of HFD feeding. In contrast, the time course of HFD-feeding did not induce significant PNN changes in a control brain area (the granular retrosplenial (RSG) cortex (Supplementary Fig. 1)), consistent with previous evidence [6]. Therefore, early in the course of DIO, at a time of accelerating weight gain, WFA+ PNN remodeling is detected selectively in the hypothalamic ARC region and exhibits a time course that parallels changes in body weight and glycemia.

**Figure 1:**
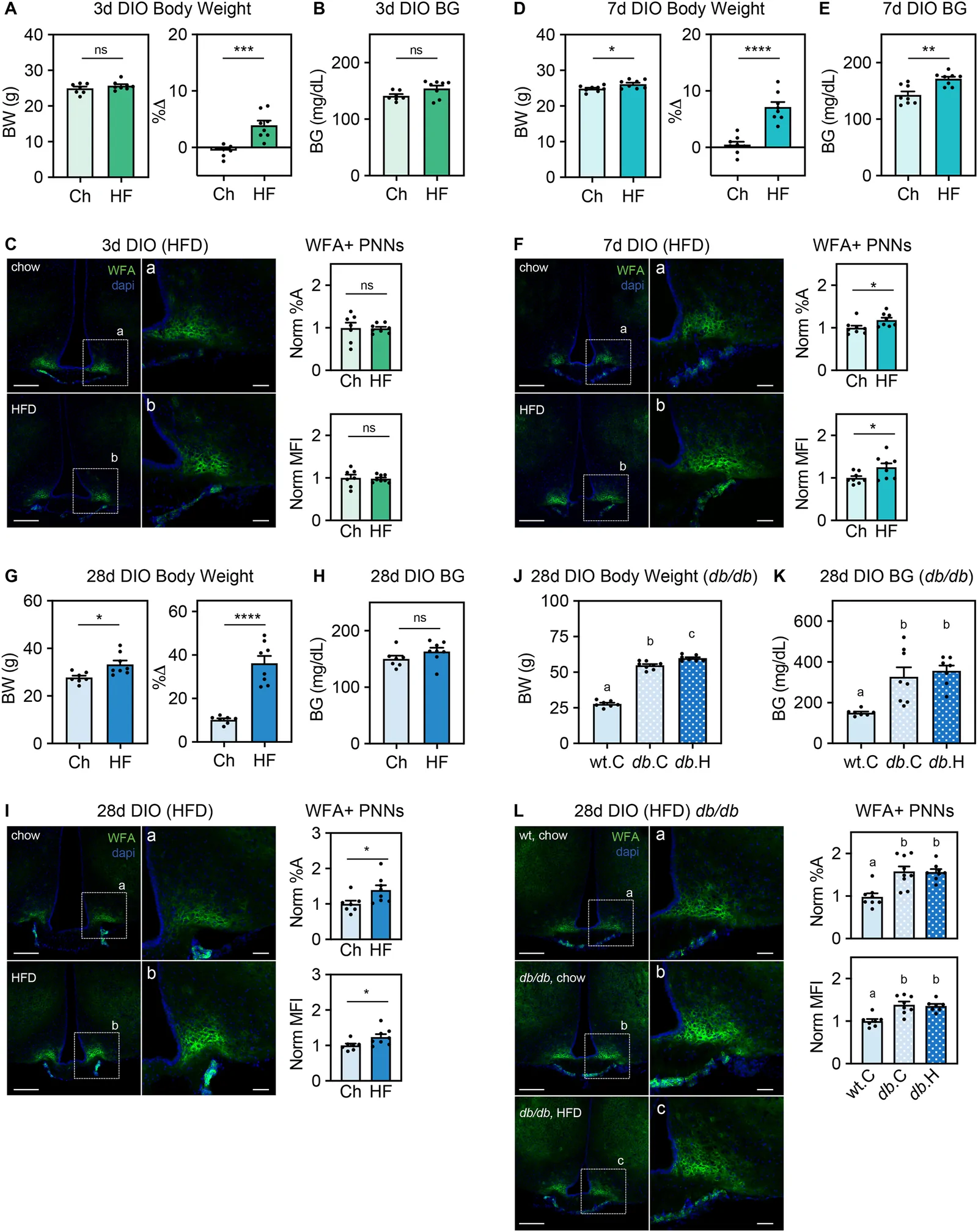
**Acute DIO increases WFA+ PNN abundance in the ARC of wild type mice.** Body weight and blood glucose in chow- and HFD-fed mice after (**A,B**) 3 d, (**D,E**) 7 d, and (**G,H**) 28 d of diet exposure. Fluorescent detection of ARC PNN CS-GAGs (using WFA) in coronal sections of mouse hypothalamus shows no difference after (**C**) 3 d but increased expression after (**F**) 7 d and (**I**) 28 d of HFD feeding compared to chow controls. Leptin-signaling deficient *db/db* mice exhibit increased (**J**) body weight and (**K**) blood glucose in association with (**L**) increased WFA+ ARC PNNs on both chow and HFD compared to wild type chow controls. Chow mice shown in G-I are also shown in J-L. Scale bar: 200 μm. (**a-c**) Higher magnification of WFA+ ARC PNN enmeshments. Scale bar: 50 μm. Data were assessed for normality using Shapiro–Wilk test; normal (Gaussian) distributed data were analyzed using either unpaired t-test (two-tailed) (⁎ indicates significance) or one-way ANOVA and Tukey's multiple comparison post-test (a,b,c indicates significance). Non-normal data was analyzed using Mann–Whitney test. Statistical analyses reported in Supplemental Tables 1,2, and 4. %A, percent area; ARC, arcuate nucleus; BG, blood glucose; BW, body weight; Ch, chow; DIO, diet-induced obesity; HF/HFD, high-fat diet; MFI, mean fluorescence intensity; PNNs, perineuronal nets; WFA, *Wisteria floribunda* agglutinin.

#### 3.1.1 HFD feeding increases body weight but not ARC WFA+ PNN abundance or glycemia in *db/db* mice

We and others have shown that in contrast to wild type mice, baseline WFA+ ARC PNN content is increased in both leptin-deficient *ob/ob* mice [4] and leptin-signaling deficient *db/db* mice on chow [6]. As this increase is detectable on a standard chow diet, an obesogenic diet does not appear to be required for ARC PNN abundance to increase, at least in the setting of an absent leptin signal. To explore this hypothesis further, we placed separate cohorts of male *db/db* mice on either chow or HFD for 28 d. We then quantified WFA+ ARC PNN content relative to wild-type controls fed chow diet (Figure 1J-L). As expected, chow-fed *db/db* mice exhibited elevated levels of body weight (Figure 1J), blood glucose (Figure 1K), and WFA+ ARC PNN abundance (Figure 1L) compared to wild type controls, consistent with a previous report [6]. Although HFD significantly increased the body weight of *db/db* mice (Figure 1J), neither blood glucose (Figure 1K) nor WFA+ ARC PNN content (Figure 1L) were further increased. In contrast, wild type mice exhibited increases of body weight, blood glucose, and ARC WFA+ PNN abundance after switching to a HFD compared to chow-fed controls (Figure 1G-I). Together, these results suggest that in *db/db* mice, leptin signaling deficiency, rather than diet composition, underlies increased ARC PNN content.

### 3.2 ARC WFA+ PNN abundance declines with chronic exposure to an obesogenic diet

Prior studies reported that after consuming a HFD [4], [12] or HFHS [6] diet for 3 months, WFA+ ARC PNN abundance either increases or does not change. Our data extend these findings by showing that after 3 m of HFD feeding, WFA+ ARC PNNs were increased in wild type mice, but the trend did not achieve statistical significance (p = 0.08) (Figure 2C). As expected, levels of both body weight (Figure 2A) and blood glucose (Figure 2B) were significantly increased at the 3-month time point compared to chow-fed controls. This evidence that HFD-induced ARC PNN remodeling evolves over time led us to ask whether the 3 m time point represents an inflection point signaling the onset of a second remodeling phase in response to long-term DIO. To test this hypothesis, we measured the effects of either HFD or HFHS exposure for time intervals ranging from 4.5 to 12 months. Unlike the earlier time points, WFA+ ARC PNN abundance declined after 4.5 m of either HFD (Figure 2D–F) or HFHS (Figure 2G–I) diet feeding, despite continued weight gain (relative to chow fed controls). This same pattern of increased body weight and reduced WFA+ ARC PNN abundance was again observed at the 12-month time point (Figure 2J,L). Blood glucose levels had also returned to baseline by this time point (Figure 2K). These data support a biphasic model in which ARC WFA+ PNNs increase early during the course of DIO, but at or around the 3-month time point, this increase has subsequently declined and returned back to baseline despite continued weight gain. In contrast, no such differences were observed in RSG cortical WFA+ PNN abundance (Supplementary Fig. 1). We also report that like WFA+ ARC PNNs, blood glucose levels exhibit an initial increase in response to HFD that reaches a steady-state between 2 and 6 m before returning towards baseline by the 12-month time point (Figure 2K).

**Figure 2:**
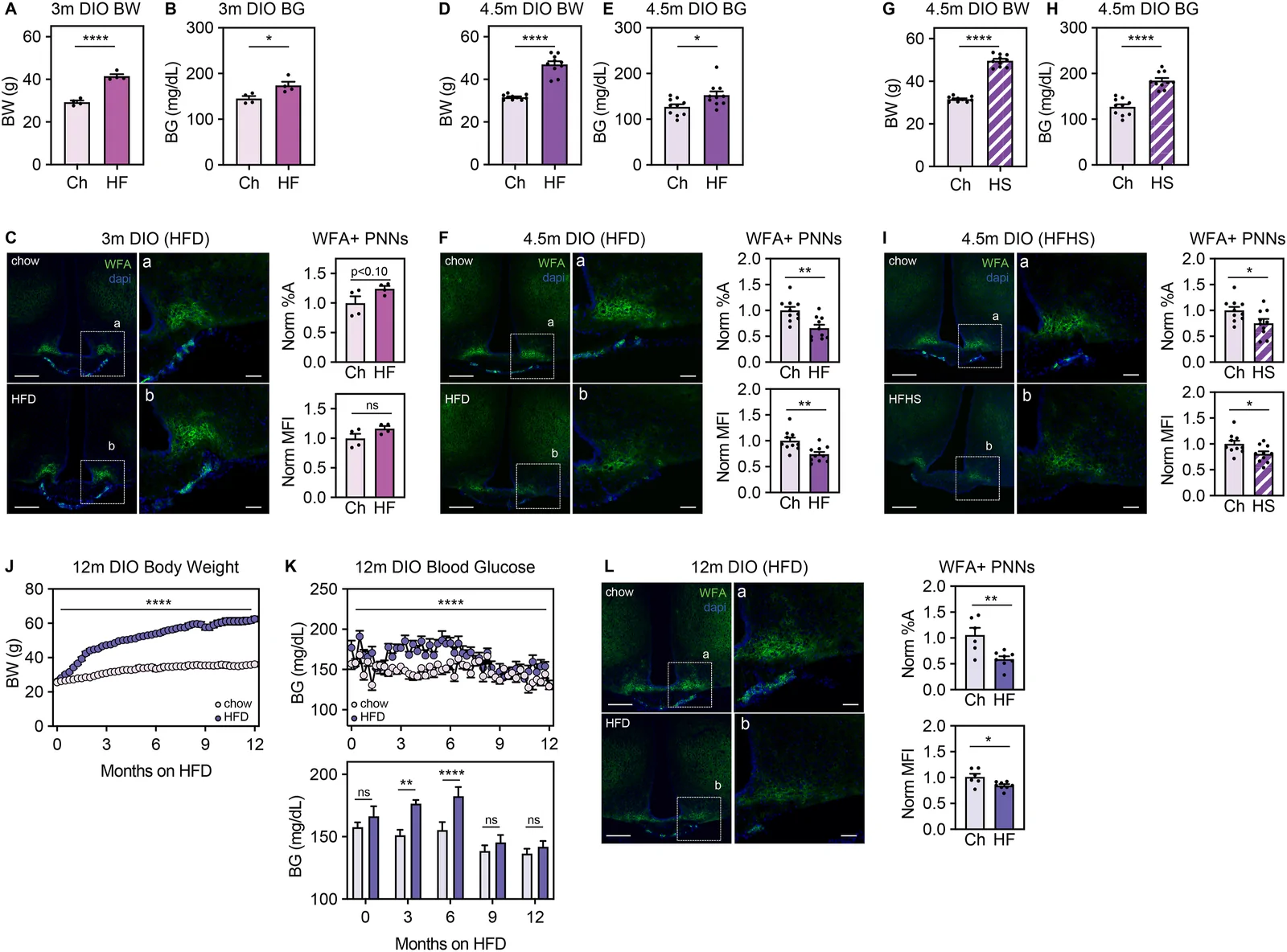
**Chronic DIO decreases WFA+ PNN abundance in the ARC of wild type mice.** Body weight and blood glucose in chow-, HFD-, and HFHS-fed mice after (**A,B**) 3 m and (**D,E,G,H**) 4.5 m of obesogenic diet exposure. Fluorescent detection of WFA+ ARC PNNs in coronal sections of mouse hypothalamus shows trending increases in WFA+ ARC PNNs after (**C**) 3 m but significantly reduced WFA+ ARC PNNs after 4.5 m (**F**) HFD and (**I**) HFHS feeding compared to chow controls. Chow mice shown in D-F are also shown in G-I. 12 m of HFD increases (**J**) body weight and (**K**) biphasic changes in blood glucose, including an initial increase followed by normalization, in response to DIO, and (**L**) reduces ARC WFA+ PNN abundance. Blood glucose shown in (K, *top*) are presented as averages between 2 weeks at each time point: 0 m (0/0.25 m), 3 m (2.75/3 m), 6 m (5.75/6 m), 9 m (8.75/9), 12 m (11.75/12) (K, *bottom*). Scale bar: 200 μm. (**a,b**) Higher magnification of WFA+ ARC PNN enmeshments. Scale bar: 50 μm. Data were assessed for normality using Shapiro–Wilk test; normal (Gaussian) distributed data were analyzed using either unpaired t-test (two-tailed) or one-way ANOVA and Tukey's multiple comparison post-test. Non-normal data was analyzed using Mann–Whitney test. Statistical analyses reported in Supplemental Tables 5–6. %A, percent area; ARC, arcuate nucleus; BG, blood glucose; BW, body weight; Ch, chow; DIO, diet-induced obesity; HF/HFD, high-fat diet; HS/HFHS, high-fat high-sucrose; MFI, mean fluorescence intensity; PNNs, perineuronal nets; WFA, *Wisteria floribunda* agglutinin.

### 3.3 ChABC pre-digestion unmasks ACAN+ ARC PNNs

An important limitation inherent in PNN analyses is that WFA+ CS-GAG attachments can hinder histological detection of the underlying Aggrecan (ACAN) protein epitope (via steric hindrance and charge interactions) [16]. To optimize detection of ACAN+ PNNs in the ARC across the DIO time course, which temporally influences the abundance of WFA+ CS-GAG labeling in this region and thus may differentially influence antibody-protein binding to the underlying ACAN core protein, we optimized an incubation time course for Chondroitinase ABC (ChABC) digestion of CS-GAG attachments prior to ACAN immunostaining (Figure 3A) (Supplementary Fig. 2). We report that ChABC pre-digestion results in a 68.6–96.3% reduction in WFA+ CS-GAG labeling in the ARC (Figure 3B,E) and yields a corresponding 1.6–1.8x increase in ACAN+ PNN detectability (Figure 3C,F). We therefore determined that ChABC pre-digestion effectively enhances histological detection of ACAN+ ARC PNNs in both chow-fed and DIO mice.

**Figure 3:**
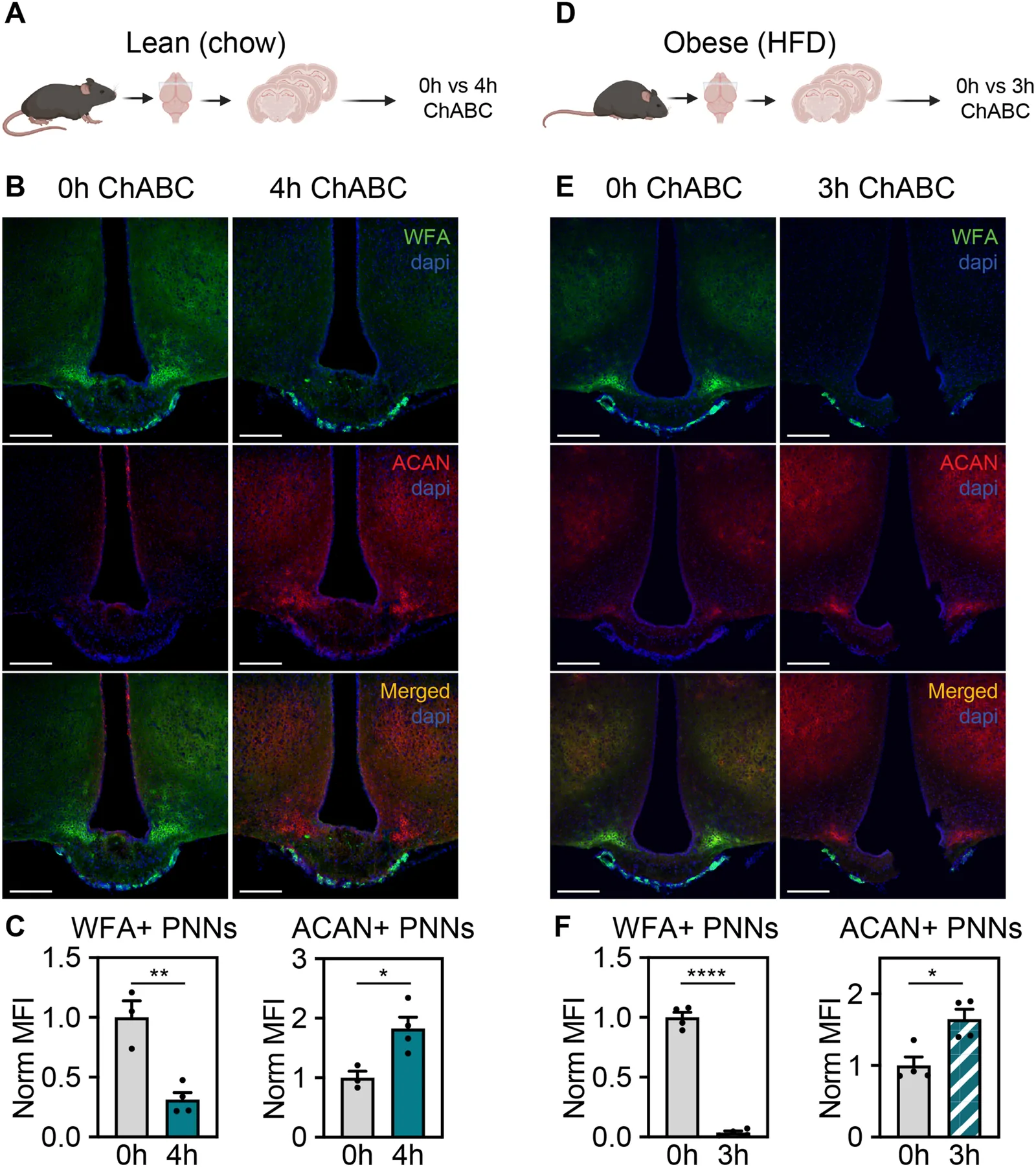
**ChABC pre-digestion of tissue CS-GAGs increases ACAN+ PNN labeling in the ARC of chow and DIO mice.** Schematic depicting technical replicates in (**A**) chow and (**D**) HFD fed mice show ChABC reduces WFA labeling of (**B,E**) ARC PNN CS-GAGs and corresponding increase in the detection of (**C,F**) ARC PNN ACAN labeling. Histology is performed in technical replicates using 3–4 sections/group. The complete time course describing the influence of ChABC pre-digestion of WFA+ and ACAN+ PNN labeling from 0 to 24 h is described in Supplementary Fig. 2. Data were assessed for normality using Shapiro–Wilk test; normal (Gaussian) distributed data were analyzed using unpaired t-test (two-tailed). Non-normal data was analyzed using Mann–Whitney test. Statistical analyses are reported in Supplemental Table 7. Scale bar: 200 μm. ACAN, aggrecan; ARC, arcuate nucleus; ChABC, Chondroitinase ABC; HFD, high-fat diet; MFI, mean fluorescence intensity; PNNs, perineuronal nets; WFA, *Wisteria floribunda* agglutinin.

### 3.4 Temporal changes in ACAN+ PNN content in the ARC of DIO mice

In contrast to the increase in WFA+ ARC PNNs observed after 7 d and 28 d HFD exposure (Figure 1), differences in ACAN+ ARC PNN expression were not observed at either time point (Figure 4A–C). After 28 d HFD feeding, for example, WFA+ PNN percent area increased 1.4-fold (Figure 1I), but no corresponding increase in the underlying ACAN core protein was observed in ChABC pre-digested tissues (Figure 4C). In comparison, we did not observe changes in either ACAN+ (Figure 4D) or WFA+ (Figure 1) ARC PNNs after 3 m of HFD. We interpret these data to suggest that early increases of WFA + PNN labeling reflect remodeling of the attached CS-GAGs rather than increased abundance of the underlying ACAN matrix structure itself. In contrast, both WFA+ and ACAN+ PNN abundance were increased in the ARC of *db/db* mice irrespective of diet (Figure 1L and Figure 4E). After long-term exposure to an obesogenic diet (both 4.5 m and 12 m), the decrease of WFA+ ARC PNNs observed in wild type mice was accompanied by reduced ACAN staining, irrespective of diet composition (Figure 4F–H). These observations support a model wherein the initial first phase, diet-induced rise of WFA+ ARC PNNs is driven primarily by increased WFA+ glycans, whereas the subsequent second phase decline with long-term DIO involves a loss of the broader PNN structure itself.

**Figure 4:**
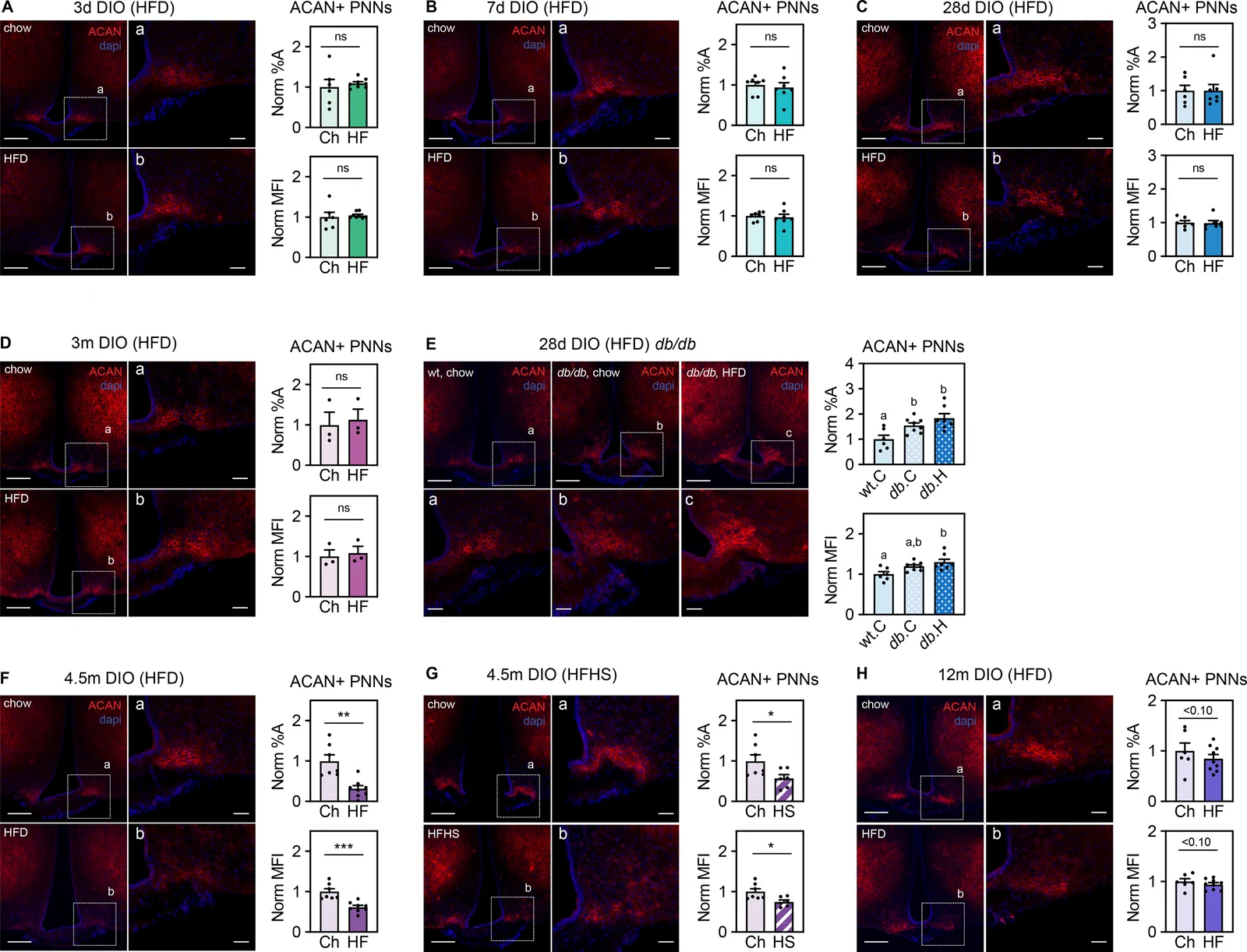
**Temporal changes in ACAN+ PNN content in the ARC of DIO mice.** ACAN+ PNN abundance in the ARC of wild type mice after (**A**) 3 d, (**B**) 7 d, (**C**) 28 d, and (**D**) 3 m of HFD feeding remains unchanged. Leptin-signaling deficient *db/db* mice exhibit (**E**) increased ARC ACAN+ PNNs on both chow and HFD conditions compared to wild type chow controls. Chow mice shown in C are also shown in E. Chronic DIO reduces ARC ACAN+ PNN abundance after (**F**) 4.5 m HFD, (**G**) 4.5 m HFHS, and (**H**) 12 m HFD feeding. Chow mice shown in F are also shown in G. Scale bar: 200 μm. (**a-c**) Higher magnification of ARC ACAN+ PNN enmeshments. Scale bar: 50 μm. Data were assessed for normality using Shapiro–Wilk test; normal (Gaussian) distributed data were analyzed using either unpaired t-test (two-tailed) (⁎ indicates significance) or one-way ANOVA and Tukey's multiple comparison post-test (a,b indicates significance). Non-normal data was analyzed using Mann–Whitney test. Statistical analyses reported in Supplemental Tables 8–9. %A, percent area; ACAN, aggrecan; ARC, arcuate nucleus; Ch, chow; DIO, diet-induced obesity; HF/HFD, high-fat diet; HS/HFHS, high-fat high-sucrose; MFI, mean fluorescence intensity; PNNs, perineuronal nets.

## 4 Discussion

Located within the ARC are neuronal populations that work in concert to promote energy and glucose homeostasis. Among these are the orexigenic AgRP and satiety-inducing anorexigenic POMC neurons, both of which are regulated by input from circulating hormones (*e.g.,* leptin, ghrelin, and insulin) and nutrients including glucose [1]. Enmeshment of these neurons by PNNs begins early after birth and is completed by postnatal Day 28, which coincides with closure of the critical period for their development [4]. In the absence of metabolic perturbation, these PNNs remain relatively stable through adulthood, functioning to constrain excitability and synaptic plasticity of the neurons they enmesh [2]. Although remodeling of PNN enmeshment has been implicated in maladaptive ARC neuron responses to obesity and diabetes [6], the temporal pattern of this remodeling process remains to be fully characterized.

In this study, we report that a significant increase of ARC PNN abundance was detectable by Day 7 after the switch from standard chow to a HFD, in association with the onset of significant weight gain (Figure 1). Our detection of increased ARC WFA+ PNNs by Day 7, which is earlier than the 14 d time point reported by *Beddows* et al. [6], warrants comment. We note that whereas weight gain was evident by Day 7 in our study, body weight did not begin to increase until Day 14 in the latter study. These observations support a model wherein the onset of ARC PNN remodeling may be triggered more by weight gain rather than by the change of diet composition. Also consistent with this model is our observation that in *db/db* mice, both obesity and increased ARC PNN abundance occur in the absence of an obesogenic diet (Figures 1 and 4). While recent work indicates that increased ARC WFA+ PNNs induced following the switch to an obesogenic diet is largely driven by reduced PNN degradation and increased expression of PNN components [6], the primary driver of this response remains unknown. One possibility is that the diet switch induces a transient increase in the activity of enmeshed AgRP neurons, which in turn stimulates PNN assembly, and additional work is warranted to test this hypothesis.

Quantifying the abundance of the underlying CSPG core protein ACAN (Figure 3) is technically challenging because the CS-GAGs that bind to WFA can hinder detection of the underlying core protein. To address this issue, we employed a ChABC pre-digestion strategy to remove the CS glycans from tissue sections prior to ACAN immunostaining [17], [18]. This approach shows that in contrast to WFA labeling, ARC ACAN+ PNN content does not increase following the switch to an obesogenic diet (either a HFD or a HFHS diet; Figure 4). Interestingly, despite increased abundance of ARC WFA+ PNNs in mice fed a HFD (Figure 1), the influence of ChABC pre-digestion on ACAN labeling was greatly enhanced in the chow group (Figure 3). One potential explanation for this finding is that in addition to altering ARC CS glycan abundance, diet composition may also impact tertiary spatial ordering and compactness of CS-GAG attachments. According to this hypothesis, the impact of ChABC digestion on ACAN antibody detection depends on both the 3D spatial organization of PNN attached CS glycans and their absolute abundance. Although a previous report showed increased ARC ACAN+ PNN immunoreactivity following the switch to an obesogenic diet [6], methodological differences offer a potential explanation for these divergent outcomes, as the earlier study did not employ ChABC pre-digestion to optimize ACAN detection across chow and DIO groups.

Taken together, our results suggest that early after the switch to an obesogenic diet, ARC WFA + PNN abundance increases despite minimal, if any, changes in ACAN core protein content. One potential explanation for this finding is that initial ARC PNN remodeling is effectively ACAN-independent, involving either hyperglycosylation of the underlying core protein or modifications to the underlying protein scaffolding. Another possibility is that additional core proteins are recruited into ARC PNNs, including Phosphacan, Versican, Neurocan, and/or Brevican [4], [6], such that WFA labeling increases independently of ACAN content.

In contrast to the early phase of DIO, the reduced abundance of ARC PNNs at later time points associate with loss of ACAN as well as attached CS-GAGs. Over time, therefore, DIO may result in reduced ARC neuron enmeshment by PNNs, and that distinct mechanisms are implicated in PNN remodeling during the early *vs.* late phase of DIO. Additional studies are warranted to test this hypothesis. Related to these findings is that the 3-month time point corresponds to the transition between the initial increase and subsequent decrease of ARC PNN abundance (Figure 2). This observation has the potential to reconcile a point of contention in the existing literature. Thus, whereas one study showed that WFA+ ARC PNN abundance increases after 3 months of consuming a HFHS diet [6], two other studies failed to show this effect [4], [12]. If the 3-month time point does indeed reflect the transition between the early and late phase of ARC PNN remodeling, as our data suggests, then it is not surprising that some studies would report an increase while others may not.

Our findings also bear on previously reported functional implications of obesity-associated changes of ARC PNN abundance. Beddows et al. [6] proposed that increased ARC PNN abundance is a form of ‘neurofibrosis’ that contributes to obesity-associated metabolic impairment. Yet we report that during chronic DIO, ARC PNN abundance decreases over time despite worsening metabolic function. Thus, factors additional to hypothalamic ‘neurofibrosis’ are implicated in the link between DIO and its long-term metabolic consequences.

To reconcile these various observations, we consider the concept that increased PNN formation is a biological response to hyperactivity of enmeshed neurons [19]. Given evidence that the PNN matrix itself is required to maintain hyperexcitability of the enmeshed neuron [9], [20], it is perhaps unsurprising that increased neuronal activity is coupled to increased PNN enmeshment. Applying this reasoning to AgRP neurons, evidence suggests that the activity of these neurons increases initially following the switch from chow to an obesogenic diet [21], [22] – a period characterized by a transient hyperphagic response – but as obesity develops over the longer term (>6 m), AgRP neuron activity is reduced [23], as has also been reported for POMC neurons [24]. Thus, we favor the hypothesis that increased ARC PNN abundance early after an obesogenic diet switch facilitates increased activity of ARC neurons, while the opposite is true later in its course. However, we acknowledge that other, non-neuronal cell types (*e.g.,* microglia, oligodendrocytes) have been linked to PNN degradation events [7], [25], [26]. Future studies that investigate glia involvement in biphasic PNN remodeling during both short- and long-term DIO, in relation to underlying neural activity, are therefore warranted.

Obesity-associated changes of glycemia also warrant comment. We report that glycemia increases in tandem with the initial phase of weight gain and elevated ARC WFA+ PNNs, and that despite ongoing weight gain, reduced ARC PNN abundance later in the course of DIO associates with the return to a more normal blood glucose level. To the extent that increased AgRP neuron activity contributes to the initial increase of glycemia, it is possible that reduced activity of these neurons helps to normalize glycemia during later phases of DIO in association with reduced ARC PNN abundance. This model is consistent with evidence that AgRP neurons are responsive to changes in the circulating glucose level [10] and that hyperactivity of these neurons is required for diabetic hyperglycemia in *ob/ob* mice [11]. Investigation into the relationships between biphasic ARC PNN remodeling, AgRP neuron activity, glucose sensing, and glucose homeostasis represents a rich opportunity for future study.

In conclusion, we report that over the course of DIO, ARC PNNs undergo biphasic remodeling characterized by an early, glycan-dominant increase followed by a reduction in the PNN matrix itself at later time points. These data add evidence that, unlike PNNs in many other brain regions, hypothalamic PNNs are dynamically responsive to acute and chronic metabolic perturbations. These changes not only track but potentially contribute to neuronal activity states [23], [24] which in turn are responsive to peripheral metabolic outputs. As such, insight into both underlying mechanisms and the consequences of these changes for neuronal control of metabolism will help to clarify the implications of hypothalamic extracellular matrix plasticity for energy and glucose homeostasis.

## CRediT authorship contribution statement

**Alexander Carbonell:** Writing – original draft, Visualization, Validation, Methodology, Formal analysis, Data curation, Conceptualization. **Jarrad M. Scarlett:** Writing – original draft, Supervision, Funding acquisition, Formal analysis, Data curation, Conceptualization. **Ellie I. James:** Writing – review & editing, Visualization, Methodology, Formal analysis, Data curation, Conceptualization. **Kendra L. Francis:** Writing – review & editing, Validation, Methodology, Formal analysis, Data curation, Conceptualization. **Kathryn Tyler:** Writing – review & editing, Validation, Methodology, Formal analysis, Data curation. **Tamim J. Chiba:** Writing – review & editing, Formal analysis, Data curation. **Xavier Xong:** Writing – review & editing, Formal analysis. **Michael A. Haidar:** Writing – review & editing, Formal analysis. **Spoorthy Siddam:** Writing – review & editing, Formal analysis, Data curation. **Aarun S. Hendrickson:** Writing – review & editing, Data curation. **Noah Marzouk:** Data curation, Writing – review & editing. **Cristiana J. Meuret:** Writing – review & editing, Data curation. **Caeley L. Bryan:** Writing – review & editing, Data curation. **Nina Brandt:** Writing – review & editing, Data curation. **Lauren E. Gilman:** Writing – review & editing, Data curation. **Jeremy M. Frey:** Writing – review & editing, Data curation. **Vladislav Baglaev:** Writing – review & editing, Data curation. **Mauricio D. Dorfman:** Writing – review & editing, Data curation, Conceptualization. **David A. Tovar:** Writing – review & editing, Formal analysis. **Gregory J. Morton:** Supervision, Funding acquisition, Conceptualization. **Joshua P. Thaler:** Writing – original draft, Supervision, Funding acquisition, Conceptualization. **Michael W. Schwartz:** Writing – original draft, Supervision, Funding acquisition, Conceptualization. **Kimberly M. Alonge:** Writing – original draft, Validation, Supervision, Methodology, Funding acquisition, Formal analysis, Data curation, Conceptualization.

## Ethics declaration

All animal husbandry and procedures were approved by the IACUC at the University of Washington (Seattle, Washington; Protocol #2456–06), performed in accordance with the National Institutes of Health Guide for the Care and Use of Laboratory Animals, and are in compliance with ARRIVE guidelines.

## Declaration of competing interest

The authors declare the following financial interests/personal relationships which may be considered as potential competing interests: Michael W. Schwartz reports relationships with Lumen Bioscience Inc, Olio Labs, and Gallant Bio that include: consulting or advisory. If there are other authors, they declare that they have no known competing financial interests or personal relationships that could have appeared to influence the work reported in this paper.

## Data Availability

Data will be made available on request.
