# Supplementary material for: Biphasic remodeling of arcuate nucleus perineuronal nets in diet-induced obese mice
Source: Mol Metab. 2026 Oct 15;114:102460. doi: 10.1016/j.molmet.2026.102460 (PMC13742481; doi:10.1016/j.molmet.2026.102460)
Supplement: Supplementary file 1 [file mmc1.pdf]

SUPPLEMENTARY MATERIALS

Biphasic remodeling of arcuate nucleus perineuronal nets in diet-induced obese mice

Alexander Carbonell[1,2†], Jarrad M. Scarlett[3,4†], Ellie I. James[3], Kendra L. Francis[3,4], Kathryn Tyler[1,2], Tamim J. Chiba[1], Xavier Xong[3], Michael A. Haidar[5,6], Spoorthy Siddam[3], Aarun S. Hendrickson[3], Noah Marzouk[1,2], Cristiana J. Meuret[5], Caeley L. Bryan[3], Nina Brandt[3], Lauren E. Gilman[1,2], Jeremy M. Frey[3], Vladislav Baglaev[3], Mauricio D. Dorfman[3], David A. Tovar[6,7], Gregory J. Morton[3], Joshua P. Thaler[3#], Michael W. Schwartz[3#], Kimberly M. Alonge[1,2,3,5#*]

[1] Department of Neuroscience, University of Florida, Gainesville, FL, USA.
[2] McKnight Brain Institute, University of Florida, Gainesville, FL, USA.
[3] University of Washington Medicine Diabetes Institute, University of Washington, Seattle, WA, USA.
[4] Department of Pediatric Gastroenterology and Hepatology, Seattle Children's Hospital, Seattle, WA, USA.
[5] Department of Medicinal Chemistry, University of Washington, Seattle, WA, USA.
[6] Data Science Institute, Vanderbilt University, Nashville, TN, USA.
[7] Department of Psychology, Vanderbilt University, Nashville, TN, USA.

[†] equal first authorship, [#] equal senior authorship

*** Corresponding Author:**
Kimberly M. Alonge, PhD
Department of Neuroscience
McKnight Brain Institute
University of Florida
1149 Newell Drive
Gainesville, FL 32610, USA
Phone: +1(352) 273-5094
Email: kimberly.alonge@ufl.edu

TABLES OF CONTENTS

Supplementary Figure 1. WFA+ PNNs in the granular retrosplenial cortex remain unchanged in response to acute and chronic DIO

Supplementary Figure 2. ChABC pre-digestion of brain tissue CS glycans enhances ACAN antibody labeling

Supplementary Table 1: Mouse measurements for 3d, 7d, and 28d chow vs HFD wild type mice (Main Figure 1)

Supplementary Table 2: WFA+ ARC PNN labeling for 3d, 7d, and 28d chow vs HFD wild type mice (Main Figure 1)

Supplementary Table 3: WFA+ RSG PNN labeling for 3d-12m chow vs HFD wild type mice (Supplementary Figure 1)

Supplementary Table 4: Mouse measurements and WFA+ ARC PNN labeling for 28d chow vs HFD in *db/db* mice (Main Figure 1)

Supplementary Table 5: Mouse measurements for 3m, 4.5m, and 12m chow vs DIO wild type mice (Main Figure 2)

Supplementary Table 6: WFA+ ARC PNN labeling for 3m, 4.5m, and 12m chow vs DIO wild type mice (Main Figure 2)

Supplementary Table 7: ARC PNN labeling after ChABC in chow vs DIO wild type mouse tissue sections (Main Figure 3 and Supplementary Figure 2)

Supplementary Table 8: ACAN+ ARC PNN histology acute and chronic DIO mice (Main Figure 4)

Supplementary Table 9: ACAN+ ARC PNN histology for 28d chow vs HFD in *db/db* mice (Main Figure 4)

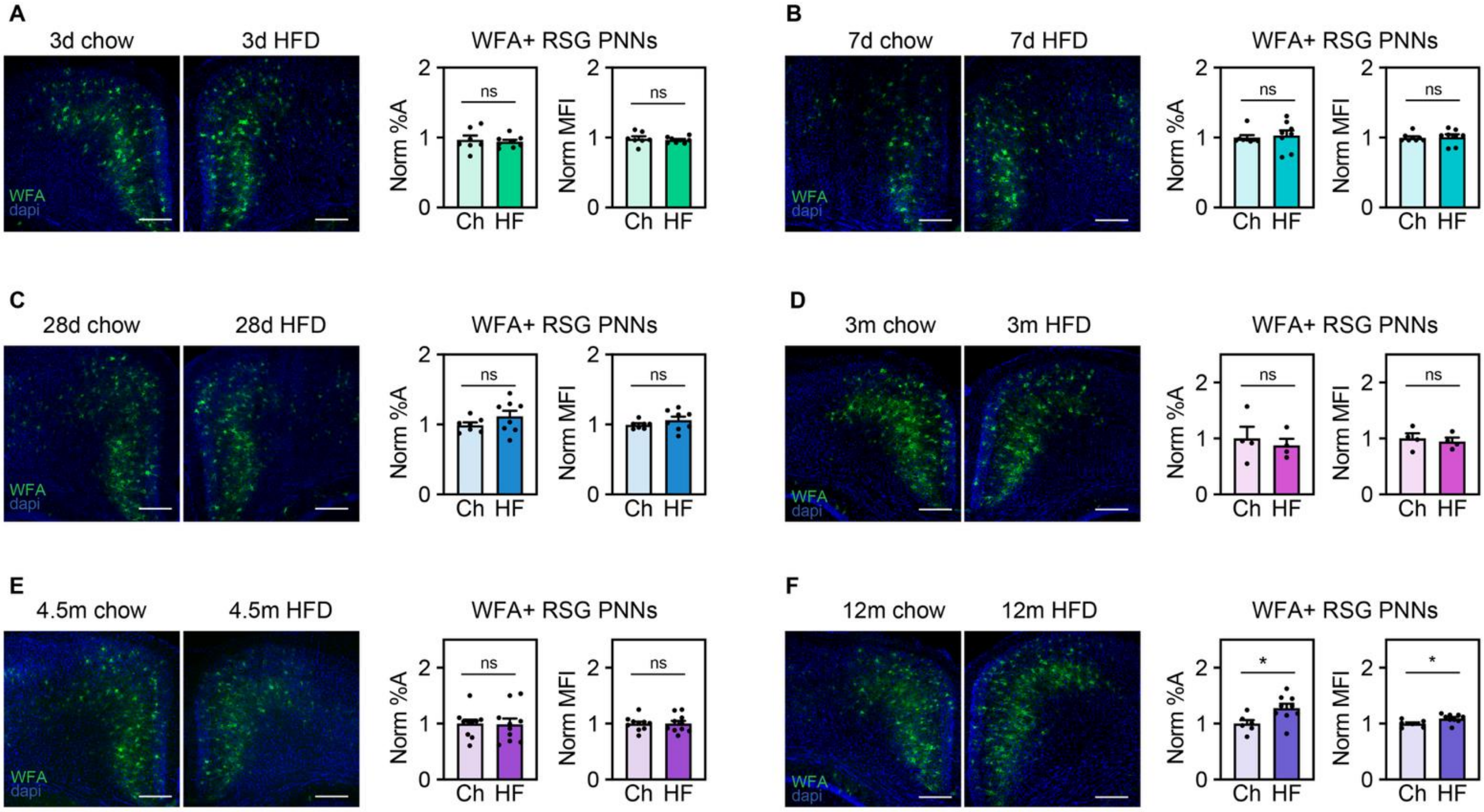

Supplementary Figure 1. WFA+ PNNs in the granular retrosplenial cortex remain unchanged in response to acute and chronic DIO. HFD doesn't influence WFA+ RSG PNN abundance after (**A**) 3d, (**B**) 7d, (**C**) 28d, (**D**) 3m, or (**E**) 4.5m of DIO. 12m of HFD feeding exhibits an (**F**) increase in WFA+ RSG PNNs, which is in contrast to the decreased expression in the ARC (main Figure 2). Scale bar: 200 µm. Data were assessed for normality using Shapiro-Wilk test; normal (Gaussian) distributed data were analyzed using unpaired t-test (two-tailed). Non-normal data was analyzed using Mann-Whitney test. Statistical analyses reported in **Supplemental Table 3**. %A, percent area; RSG, granular retrosplenial cortex; HF/HFD, high-fat diet; MFI, mean fluorescence intensity; PNNs, perineuronal nets; WFA, *Wisteria floribunda* agglutinin.

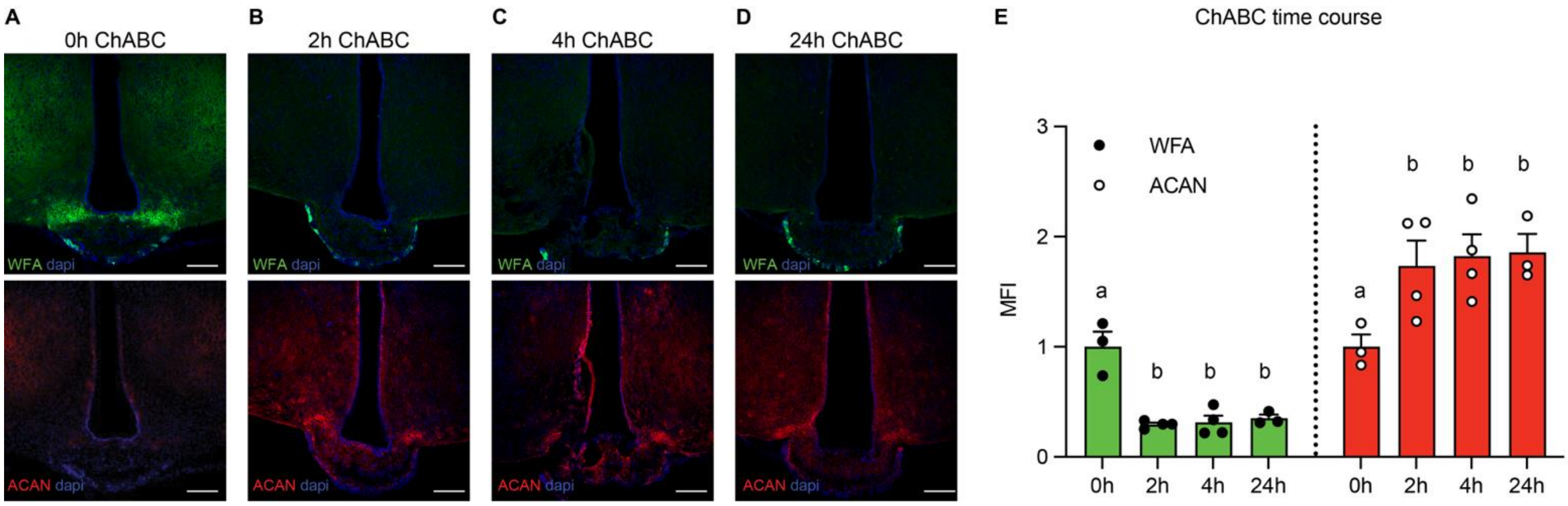

Supplementary Figure 2. ChABC pre-digestion of brain tissue CS glycans enhances ACAN antibody labeling. Compared to (**A**) non-digested controls, (**B**) 2h of ChABC pre-digestion of brain tissue sections of a chow-fed mouse shows loss of WFA+ CS-GAGs associates with enhanced antibody detection of ACAN, which was comparable to (**C**) 4h and (**D**) 24h digestion time points, quantified in (**E**). Scale bar: 200 µm. Data were assessed for normality using Shapiro-Wilk test; normal (Gaussian) distributed data were analyzed using a 2-way anova with matched PNN targets and Tukey multiple comparisons. Statistical analyses reported in **Supplemental Table 7**. ACAN, aggrecan; ChABC, chondroitinaseABC; MFI, mean fluorescence intensity; PNN, perineuronal net; WFA, *Wisteria floribunda* agglutinin.

Supplementary Table 1: Mouse measurements for 3d, 7d, and 28d chow vs HFD wild type mice (Main Figure 1)			
**t-test** p-value (t, df)<br>**Normality** p-value (chow, HFD)	**Body weight (g)**	**Body weight (%Δ)**	**Blood Glucose (mg/dL)**
**3d HFD**<br>(n=7 chow, n=8 HFD)	0.29 (1.10, 13)<br>**Yes** (0.35, 0.25)	**0.0005*** (4.60, 13)<br>**Yes** (0.47, 0.59)	0.14<br>**No** (0.44, 0.03)
**7d HFD**<br>(n=8 chow, n=8 HFD)	**0.024*** (2.53, 14)<br>**Yes** (0.94, 0.12)	**<0.0001*** (6.26, 14)<br>**Yes** (0.96, 0.77)	**0.0016*** (3.91, 14)<br>**Yes** (0.11, 0.31)
**28d HFD**<br>(n=7 chow, n=8 HFD)	**0.012*** (2.94, 13)<br>**Yes** (0.89, 0.12)	**<0.0001*** (7.14, 13)<br>**Yes** (0.57, 0.24)	0.18 (1.41, 13)<br>**Yes** (0.41, 0.14)
Statistics: *Data were assessed for normality using Shapiro-Wilk test; normal (Gaussian) distributed data were analyzed using unpaired t-test (two-tailed). Non-normal data was analyzed using Mann-Whitney test.*			

Supplementary Table 2: WFA+ ARC PNN labeling for 3d, 7d, and 28d chow vs HFD wild type mice (Main Figure 1)		
**t-test** p-value (t, df)<br>**Normality** p-value (chow, HFD)	**WFA (MFI)**	**WFA (% area)**
**3d HFD**<br>(n=7 chow, n=8 HFD)	0.83 (0.22, 13)<br>**Yes** (0.84, 0.19)	0.92 (0.10, 13)<br>**Yes** (0.60, 0.54)
**7d HFD**<br>(n=8 chow, n=8 HFD)	**0.035*** (2.33, 14)<br>**Yes** (0.30, 0.44)	**0.033*** (2.36, 14)<br>**Yes** (0.06, 0.29)
**28d HFD**<br>(n=7 chow, n=8 HFD)	**0.040*** (2.29, 13)<br>**Yes** (0.43, 0.41)	**0.035*** (2.36, 13)<br>**Yes** (0.41, 0.28)
Statistics: *Data were assessed for normality using Shapiro-Wilk test; normal (Gaussian) distributed data were analyzed using unpaired t-test (two-tailed).*		

Supplementary Table 3: WFA+ RSG PNN labeling for 3d-12m chow vs HFD wild type mice (Supplementary Figure 1)		
**t-test** p-value (t, df)<br>**Normality** p-value (chow, HFD)	**WFA (MFI)**	**WFA (% area)**
**3d HFD**<br>(n=7 chow, n=8 HFD)	0.59 (0.55, 13)<br>**Yes** (0.45, 0.53)	0.69 (0.41, 13)<br>**Yes** (0.54, 0.72)
**7d HFD**<br>(n=8 chow, n=8 HFD)	0.27<br>**No** (0.01, 0.11)	0.27<br>**No** (<0.0001, 0.54)
**28d HFD**<br>(n=7 chow, n=8 HFD)	0.26 (1.18, 13)<br>**Yes** (0.23, 0.72)	0.20 (1.35, 13)<br>**Yes** (0.76, 0.67)
**3m HFD**<br>(n=4 chow, n=4 HFD)	0.68 (0.43, 6)<br>**Yes** (0.89, 0.82)	0.63 (0.50, 6)<br>**Yes** (0.61, 0.56)
**4.5m HFD**<br>(n=10 chow, n=10 HFD)	0.97 (0.04, 18)<br>**Yes** (0.65, 0.39)	0.93 (0.08, 18)<br>**Yes** (0.25, 0.19)
**12m HFD**<br>(n=6 chow, n=9 HFD)	**0.027*** (2.49, 13)<br>**Yes** (0.73, 0.25)	**0.024*** (2.56, 13)<br>**Yes** (0.99, 0.75)
Statistics: *Data were assessed for normality using Shapiro-Wilk test; normal (Gaussian) distributed data were analyzed using unpaired t-test (two-tailed). Non-normal data was analyzed using Mann-Whitney test. Due to technical errors in histology processing and/or missing sections: 12m HFD (n=3 chow) mice were excluded from the analyses.*		

Supplementary Table 4: Mouse measurements and WFA+ ARC PNN labeling for 28d chow vs HFD in *db/db* mice (Main Figure 1)			
**One-way ANOVA w/ Tukey's multiple comparison**<br><br>**p-value**<br>F (DFn, DFd)<br>**Normality p-value** (wt-C, Lep$^{db/db}$ -C, Lep$^{db/db}$ -H)	**wt** (chow) vs ***db/db*** (chow)	**wt** (chow) vs ***db/db*** (HFD)	***db/db*** (chow) vs ***db/db*** (HFD)
	**Body weight (g)**		
**<0.0001***<br>358.6 (2, 19)<br>**Yes** (0.89, 0.75, 0.14)	**<0.0001***	**<0.0001***	**0.0022***
	**Blood Glucose (mg/dL)**		
**0.0007***<br>10.91 (2, 19)<br>**Yes** (0.41, 0.22, 0.30)	**0.0033***	**0.0011***	0.81
	**WFA (MFI)**		
**0.0004***<br>11.92 (2, 19)<br>**Yes** (0.43, 0.52, 0.76)	**0.0007***	**0.0022***	0.93
	**WFA (% area)**		
**0.0003***<br>12.32 (2, 22)<br>**Yes** (0.18, 0.26, 0.76)	**0.0006***	**0.0010***	>0.99

Statistics: n=7 wt chow, n=8 db/db chow, n=7 db/db HFD mice. Data were assessed for normality using Shapiro -Wilk test; normal (Gaussian) distributed data were analyzed using one-way ANOVA and Tukey's multiple comparison post-test.

Supplementary Table 5: Mouse measurements for 3m, 4.5m, and 12m chow vs DIO wild type mice (Main Figure 2)			
**t-test** p-value (t, df)<br>**Normality** p-value (chow, HFD)	**Body weight (g)**		**Blood Glucose (mg/dL)**
**3m HFD**<br>(n=4 chow, n=4 HFD)	**<0.0001*** (9.15, 6)<br>**Yes** (0.91, 0.07)		**0.029*** (2.87, 6)<br>**Yes** (0.23, 0.34)
**4.5m HFD**<br>(n=10 chow, n=10 HFD)	**<0.0001*** (10.22, 18)<br>**Yes** (0.56, 0.30)		**0.019*** (2.58, 18)<br>**Yes** (0.64, 0.12)
**4.5m HFHS**<br>(n=10 chow, n=10 HFHS)	**<0.0001*** (18.89, 18)<br>**Yes** (0.56, 0.50)		**<0.0001*** (7.09, 18)<br>**Yes** (0.64, 0.29)
**12m HFD**<br>(n=9 chow, n=9 HFD)			
**2-way ANOVA**	**Body weight (g)**	**Blood Glucose (mg/dL)**	**Blood Glucose (mg/dL) 5 points**
**2-way ANOVA**<br>p-value<br>F (DFn, DFd)	**<0.0001***<br>14.84 (1.87, 29.82)	**<0.0001***<br>29.10 (1, 16)	**0.0002***<br>22.85 (1, 16)<br>Ch vs HF (p-value): 0m (0.35)**, 3m** (**0.0017***),<br>**6m** (<**0.0001***), 9m (0.87), 12m (0.99)

Statistics: *Data were assessed for normality using Shapiro-Wilk test; normal (Gaussian) distributed data were analyzed using unpaired t-test (two-tailed). For the 12m DIO study, body measurements were assessed starting from Day 0 using 2-way ANOVA or Mixed-effects full model (time x treatment; body weight) or single model (treatment alone; blood glucose) with Sidak's multiple comparison test.*

Supplementary Table 6: WFA+ ARC PNN labeling for 3m, 4.5m, and 12m chow vs DIO wild type mice (Main Figure 2)		
**t-test** p-value (t, df)<br>**Normality** p-value (chow, HFD or HFHS)	**WFA (MFI)**	**WFA (% area)**
**3m HFD**<br>(n=4 chow, n=4 HFD)	0.12 (1.83, 6)<br>**Yes** (0.24, 0.25)	<0.10 (1.96, 6)<br>**Yes** (0.11, 0.53)
**4.5m HFD**<br>(n=10 chow, n=10 HFD)	**0.0037*** (3.34, 18)<br>**Yes** (0.43, 0.52)	**0.0024*** (3.53, 18)<br>**Yes** (0.75, 0.19)
**4.5m HFHS**<br>(n=10 chow, n=10 HFD)	**0.029*** (2.38, 18)<br>**Yes** (0.43, 0.87)	**0.034*** (2.30, 18)<br>**Yes** (0.75, 0.82)
**12m HFD**<br>(n=6 chow, n=9 HFD)	**0.016*** (2.78, 13)<br>**Yes** (0.58, 0.28)	**0.0031*** (3.61, 13)<br>**Yes** (0.68, 0.26)

Statistics: *Data were assessed for normality using Shapiro-Wilk test; normal (Gaussian) distributed data were analyzed using unpaired t-test (two-tailed). Due to technical errors in histology processing and/or missing sections: 12m HFD (n=3 chow) were excluded from the analysis.*

Supplementary Table 7: ARC PNN labeling after ChABC in chow vs DIO wild type mouse tissue sections (Main Figure 3 and Supplementary Figure 2)						
**Supplementary Figure 2: ChABC pre-digestion time course establishing loss of ARC WFA+ PNN, and gain of ARC ACAN+ PNN, labeling in chow mice**						
**One-way ANOVA w/ Tukey's multiple comparison test**<br><br>**p-value**<br>F (DFn, DFd)<br>**Normality p-value** (0h, 2h, 4h, 24h)	**0h vs 2h**	**0h vs 4h**	**0h vs 24h**	**2h vs 4h**	**2h vs 24h**	**4h vs 24h**
**0.0010***<br>12.48 (3, 10)<br>**WFA:**<br>**Yes** (0.64, 0.67, 0.29, 0.16)<br>**ACAN:**<br>**Yes** (0.57, 0.18, 0.88, 0.29)	**WFA: 0.015***<br>**ACAN: 0.011***	**WFA: 0.018***<br>**ACAN: 0.0042***	**WFA: 0.041***<br>**ACAN: 0.0053***	**WFA:** >0.99<br>**ACAN:** 0.97	**WFA:** 0.99<br>**ACAN:** 0.94	**WFA:** >0.99<br>**ACAN:** >0.99

**Main Figure 3: Single time point showing effects of ChABC pre-digestion on WFA+ and ACAN+ PNN labeling in chow and HFD mice**			
**t-test** p-value (t, df)<br>**Normality** p-value (0h, 3h/4h)	**WFA (MFI)**	**ACAN (MFI)**	
**chow 0h vs 4h**	**0.0040*** (5.02, 5)<br>**Yes** (0.64, 0.29)	**0.022*** (3.28, 5)<br>**Yes** (0.57, 0.88)	
**HFD 0h vs 3h**	**<0.0001*** (20.22, 6)<br>**Yes** (0.41, 0.55)	**0.029***<br>**No** (0.02, <0.05)	

Statistics: *Chow time points (0h, n=3; 2h, n=4; 4h, n=4; 24h, n=3 sections) and HFD time points (0h, n=4; 3h, n=4 sections). Data were assessed for normality using Shapiro-Wilk test; normal (Gaussian) distributed data were analyzed using either a 2-way anova, PNN matched, full model w/ Tukey multiple comparisons (time course), or unpaired t-test (two-tailed) for the single time point comparison. Non-normal data was analyzed using Mann-Whitney test.*

**Supplementary Table 8: ACAN+ ARC PNN histology in acute and chronic wild type DIO mice (Main Figure 4)**		
**t-test** p-value (t, df)<br>**Normality** p-value (chow, HFD or HFHS)	**ACAN (MFI)**	**ACAN (% area)**
**3d HFD**<br>(n=6 chow, n=8 HFD)	0.35<br>**No** (0.41, 0.03)	0.59 (0.55, 12)<br>**Yes** (0.80, 0.73)
**7d HFD**<br>(n=7 chow, n=7 HFD)	0.74 (0.34, 12)<br>**Yes** (0.09, 0.78)	0.71<br>**No** (0.04, 0.80)
**28d HFD**<br>(n=6 chow, n=7 HFD)	>0.99<br>**No** (0.79, 0.02)	>0.99<br>**No** (0.55, 0.01)
**3m HFD**<br>(n=3 chow, n=3 HFD)	0.72 (0.38, 4)<br>**Yes** (0.22, 0.23)	0.77 (0.31, 4)<br>**Yes** (0.27, 0.24)
**4.5m HFD**<br>(n=7 chow, n=8 HFD)	**0.0006*** (4.53, 13)<br>**Yes** (0.09, 0.99)	**0.0011*** (4.16, 13)<br>**Yes** (0.10, 0.68)
**4.5m HFHS**<br>(n=7 chow, n=6 HFHS)	**0.021*** (2.70, 11)<br>**Yes** (0.09, 0.55)	**0.043*** (2.29, 11)<br>**Yes** (0.10, 0.37)
**12m HFD**<br>(n=9 chow, n=9 HFD)	0.083 (1.85, 16)<br>**Yes** (0.85, 0.42)	0.085 (1.84, 16)<br>**Yes** (0.24, 0.57)

Statistics: *Data were assessed for normality using Shapiro-Wilk test; normal (Gaussian) distributed data were analyzed using unpaired t-test (two-tailed). Non-normal data was analyzed using Mann-Whitney test. Due to technical errors in processing and/or missing sections: 3d HFD (n=1 chow), 7d HFD (n=1 chow, n=1 HFD), 28d HFD (n=1 chow, n=1 HFD), 3m HFD (n=1 chow, n=1 HFD), 4.5m HFD/HFHS (n=3 chow, n=2 HFD, n=4 HFHS) were excluded.*

**Supplementary Table 9: ACAN+ ARC PNN histology for 28d chow vs HFD in *db/db* mice (Main Figure 4)**			
**One-way ANOVA w/ Tukey's multiple comparison test**<br><br>**p-value**<br>F (DFn, DFd)<br>**Normality p-value** (wt-C, $Lep^{db/db}$-C, $Lep^{db/db}$-H)	**wt** (chow)<br>**vs *db/db*** (chow)	**wt** (chow)<br>**vs *db/db*** (HFD)	***db/db*** (chow)<br>**vs *db/db*** (HFD)
		**ACAN (MFI)**	
**0.012***<br>5.79 (2, 18)<br>**Yes** (0.79, 0.93, 0.11)	0.085	**0.0092***	0.44
		**ACAN (% area)**	
**0.0044***<br>7.47 (2, 18)<br>**Yes** (0.55, 0.85, 0.29)	**0.048***	**0.0034***	0.35

Statistics: n=6 wt chow, n=8 db/db chow, n=7 db/db HFD mice. Data were assessed for normality using Shapiro-Wilk test; normal (Gaussian) distributed data were analyzed using one-way ANOVA and Tukey's multiple comparison post-test. Due to technical errors in histology processing and/or missing sections: n=1 wt chow mouse was excluded from the (+ChABC) analyses.
